# Evaluation of an Artificial Intelligence (AI) Simulated Virtual Patient Platform for Psychiatry Communication Skills Training: A Pilot Study

**DOI:** 10.1192/bjo.2026.11161

**Published:** 2026-06-30

**Authors:** Athanasios Hassoulas, Benjamin Williams, Aatreya Sugur, Lucy Morris, Jon Turvey

**Affiliations:** 1 Cardiff University, Cardiff, United Kingdom; 2 SimFlow.AI, London, United Kingdom

## Abstract

**Aims:**

Effective communication and comprehensive history taking are core competencies in undergraduate medical education. Emerging digital technologies, including generative artificial intelligence (GenAI), offer novel opportunities to enhance experiential learning in this domain. This service evaluation explored the educational value of a GenAI-simulated virtual patient presenting with psychosis, integrated into psychiatry communication skillsteaching at Cardiff University School of Medicine. The primary aim was to assess student perceptions of the simulation as a learning tool.

**Methods:**

A mixed-methods service evaluation design was employed. Quantitative and qualitative data were collected using pre- and post-intervention questionnaires assessing self-reported confidence in communication, history taking, and risk assessment, alongside usability and accessibility. Participants engaged with an AI-simulated virtual patient on the SimFlow.AI platform, receiving pre-task information and automated post-task feedback on performance. The simulation involved an acute psychotic presentation, requiring participants to conduct a psychiatric history and risk assessment. Participants were recruited from Years 2 through to 5 of the undergraduate medical programme at Cardiff University. The AI simulation was also embedded in a Year 2 unit of study to support students’ self-directed practice of communication skills.

**Results:**

Twenty participants completed all study components, including full engagement with the AI simulation and pre- and post-intervention questionnaires. Self-reported confidence in history taking and risk assessment increased significantly following exposure to the AI-simulated virtual patient (z=−3.750, *p*<0.001), with significant improvements observed across both junior and senior cohorts. All participants reported that the simulation facilitated clinically relevant skills practice, with 94% indicating that the platform helped them identify strengths and areas for improvement in their communication skills. Perceived authenticity was high, with 94% rating the AI responses as realistic and clinically plausible. All participants endorsed the platform as a useful adjunct to existing communication skills teaching. Where the AI simulation was embedded within a Year 2 unit of study, 74% of 93 respondents rated the AI virtual patient encounter as useful in practicing psychiatric history taking during self-directed learning time.

**Conclusion:**

AI-simulated psychosis consultations appear to offer psychologically safe, accessible, and practical opportunities for communication skills rehearsal and reflective learning. Students supported the integration of AI simulations as a supplementary educational resource (particularly for OSCE/ISCE preparation) while emphasising that such tools should complement rather than replace patient actors.

